# Rapid genetic targeting of pial surface neural progenitors and immature neurons by neonatal electroporation

**DOI:** 10.1186/1749-8104-7-26

**Published:** 2012-07-10

**Authors:** Joshua J Breunig, David Gate, Rachelle Levy, Javier Rodriguez, Gi Bum Kim, Moise Danielpour, Clive N Svendsen, Terrence Town

**Affiliations:** 1 Regenerative Medicine Institute, SSB 345, Los Angeles, CA, 90048, USA; 2 Department of Biomedical Sciences, Cedars-Sinai Medical Center, Los Angeles, CA, 90048, USA; 3 Department of Neurosurgery, Cedars-Sinai Medical Center, Los Angeles, CA, 90048, USA; 4 Department of Medicine, David Geffen School of Medicine, University of California Los Angeles, Los Angeles, CA, 90048, USA

## Abstract

**Background:**

Recent findings have indicated the presence of a progenitor domain at the marginal zone/layer 1 of the cerebral cortex, and it has been suggested that these progenitors have neurogenic and gliogenic potential. However, their contribution to the histogenesis of the cortex remains poorly understood due to difficulties associated with genetically manipulating these unique cells in a population-specific manner.

**Results:**

We have adapted the electroporation technique to target pial surface cells for rapid genetic manipulation at postnatal day 2. *In vivo* data show that most of these cells proliferate and progressively differentiate into both neuronal and glial subtypes. Furthermore, these cells localize to the superficial layers of the optic tectum and cerebral cortex prior to migration away from the surface.

**Conclusions:**

We provide a foundation upon which future studies can begin to elucidate the molecular controls governing neural progenitor fate, migration, differentiation, and contribution to cortical and tectal histogenesis. Furthermore, specific genetic targeting of such neural progenitor populations will likely be of future clinical interest.

## Background

Beginning during embryogenesis and proceeding onward, the pattern of neurogenesis largely consists of neurons or neural progenitors born from asymmetric divisions of neuroepithelial cells along the neural tube (reviewed in [1]). Later on during early neurogenesis, these neuroepithelial cells convert to radial glia, but cell birth and migration generally continues to follow an inside-out pattern, with a few notable exceptions [1]. In the human brain, for example, the earliest neurons appear to migrate tangentially from the subpallium [2]. Another example is the tangential migration of rhombic lip progenitors to the external granule layer (EGL) of the cerebellum [3]. In the EGL, these progenitors continue to divide before migrating inwards and subsequently differentiating into mature granule cells in the internal granule layer. Additionally, interneurons of the cerebral cortex and olfactory bulb also display a largely tangential mode of migration [4]. In the case of cortical interneurons, these progenitors travel through several telencephalic compartments following their birth in the medial ganglionic eminence. Migrating interneurons can be found in the subventricular zone (SVZ) [5], the intermediate zone (IZ) [6], and the marginal zone (MZ) [7-9]. Notably, in the MZ, it has been shown that these interneurons arise from three distinct sources, seemingly displaying unique responses to local cues or responding to different sets of cues, as each population has different migration dynamics [7].

The marginal zone/layer 1 of the brain has recently become more appreciated as a site of neurogenesis and gliogenesis [10,11]. Cell proliferation in this region occurs starting at the earliest time of neurogenesis [2], and even in the adult, neurogenesis has been reported to occur following hypoxia [11]. Interestingly, this progenitor zone expands in both laminin γ1 knockout mice and Pax6 mutant mice [10]. However, the above studies focused on disparate paradigms of embryonic neurogenesis and post-injury adult neurogenesis, and cell autonomous molecular investigation has been extremely difficult. Accordingly, the relative size and contribution of the postnatal marginal zone to histogenesis in the dorsal compartments of the brain remains under-explored. Specifically, it is not known whether interneurons are continually proliferating at the pial surface during the postnatal period. Moreover, the relative *in vivo* potential of this progenitor region remains unexplored due to lack of methods for facile genetic manipulation of these cells in a temporally and spatially constrained manner.

One such method that holds promise as a rapid delivery system for multiple genes simultaneously is electroporation (EP). EP is defined as the use of an electric current to drive charged macromolecules such as plasmid DNA into eukaryotic cells (reviewed in [12,13]). EP of plasmids has been widely adopted as a tool for the rapid *in vivo* genetic manipulation of neural stem cells [12]. Initially, it was used for *in utero* manipulation of radial glia surrounding the lateral ventricles of the mouse embryo [13]. More recently, this technique has been extended for use in postnatal and adult brains [14-16]. However, except for one report in the hippocampus [14], most uses of EP to study neural stem cells involve injection of plasmid DNA into the brain ventricles.

We have adapted the EP technique to specifically and reproducibly target pial surface cells in multiple brain regions at postnatal day 2 (P2). Using this technique, we show that progenitors can be labeled and that they subsequently differentiate into neurons and astrocytes. This study builds a framework for future investigations aimed at studying the dynamics and molecular mechanisms of differentiation in this unique progenitor zone.

## Results

### Electroporation of the dorsal surface of the cerebral cortex

**Figure 1:**
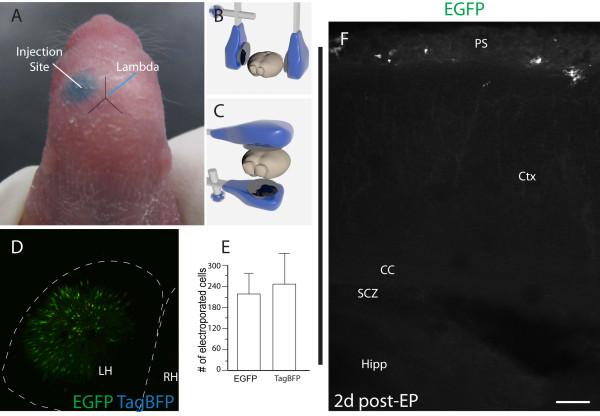
**Electroporation of the superficial perinatal cortex.** (**A**) After identifying lambda through the surface of the skull, an injection was made roughly equidistant between lambda and the eye bulge. (**B**) Parallel orientation of tweezertrodes for cortical electroporation (EP). Note that the positive electrode is placed on the right side of the skull to drive negatively-charged plasmid DNA in this direction. (**C**) Orientation of tweezertrodes for directing current toward the ventral side. (**D**) Maximum intensity projection of the cortical pial surface showing EGFP (green) and nuclear TagBFP (actually blue, but pseudocolored red) as imaged with a macroconfocal microscope. (**E**) Quantification of electroporated cells (*n* = 3 mouse pups each). (**F**) EGFP^+^ cells localize to the superficial layers and pial surface at 2d post-EP. Note that EGFP^+^ radial processes are not observed. Scale bar measures 100 μm in (**F**).

During the course of previous lateral ventricle EP studies, we occasionally detected electroporated cells at the pial surface of the cerebral cortex (J.J.B., personal observation). To more directly target this population, we delivered plasmid DNA into the meningeal space overlying the superficial layers/marginal zone of the cortex at P2. A pulled glass capillary tube was used to carefully puncture the skull and overlying skin. The tube was inserted until no resistance was felt from the skull. Care was taken to avoid penetrating the pial surface, and the plasmid DNA solution was found to evenly spread over the surface of the brain. This method is ‘tunable’, as the pulse width and volume can be adjusted to modulate velocity (and therefore force) of the injection volume delivered. With care, disturbance of the cerebral surface can be avoided, although we would occasionally notice a hematoma due to trauma to the vasculature. If the tip was too superficial, the DNA solution would immediately escape out of the injection site. Conversely, injections which are too deep do not evenly spread across the surface of the brain. A diagram showing the procedure is shown in Additional file 1: Figure S1, and the result of a successful injection is shown in Figure 1A, where the injected plasmid DNA (as labeled with fast green dye) dispersed over the entire pial surface.

To fine-tune the method, we attempted several different orientations of the tweezertrodes (Figure 1B, C). The first orientation directed current roughly parallel to the pial surface (though the surface is curved, so the EP vector is somewhat tangential). This allowed for successful EP of cells on the cortical surface (Figure 1D). Furthermore, we directed the current in a downward vector and observed a similar abundance of electroporated cells. (The parallel orientation was used for most experiments.) Electroporated cell counts with two different reporter plasmids (cytoplasmic EGFP and nuclear TagBFP) revealed signals that mostly colocalized (Figure 1D), and similar numbers of labeled cells (Figure 1E) were identified. Each label occupied the same general surface area (2.7 ± 0.64 mm^2^; *n* = 3). One concern might be that EP of radial glial end-feet occurred in lieu of pial surface cells, and that these cells may then convert into neural cells at the pial surface. However, we did not observe radial glial labeling two days post-EP using the present methodology (Figure 1F). In addition, when DNA solution was injected into the ventricle and electroporated with the same electrode orientation used for pial surface EP, our method did not label radial units associated with the medial cortex (Additional file 2: Figure S2A-A^4^). Instead, this approach resulted in labeling of radial glia that oriented: (1) inwards toward the hippocampus; or (2) towards the midline (Additional file 2: Figure S2A^4^).

### Spatially confined electroporation of the tectum pial surface

**Figure 2:**
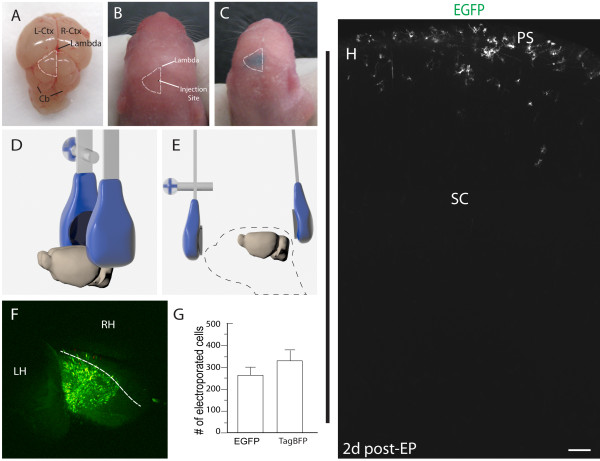
**Perinatal superficial tectum electroporation.** (**A**) An *en face* preparation is shown of the perinatal mouse brain. The left optic tectum (that is, superior colliculus) is outlined in white. (**B)** A representative image of a day 2 postnatal mouse pup is shown prior to injection into the superior colliculus. (**C**) A day 2 postnatal mouse pup injected with plasmid DNA and fast green dye is shown, highlighting unilateral labeling of the tectal surface. Images depicting (**D**) parallel or (**E**) forward-directed electrode orientation are shown. (**F**) A maximum intensity projection of the superior colliculus is shown depicting EGFP (green) and nuclear TagBFP (actually blue, but pseudocolored red) as imaged with a macroconfocal microscope. (**G**) Quantification of electroporated cells (*n* = 3 mouse pups each) is shown. (**H**) Electroporated EGFP labels a cohort of cell types at the pial surface. Scale bar measures 100 μm in H.

Given our results in the cortex, we sought to extend our findings to other superficial cerebral regions. The optic tectum (also known as the superior colliculus; Figure 2A) is readily identifiable through the perinatal skull (Figure 2B), and we found that targeting this brain area by free-hand injection was facile (Figure 2C). In fact, the injected solution often perfectly filled the entire surface of the tectum on one side, likely being limited by the overhang of the posterior cortex on its anterior aspect and by the inferior colliculus at the posterior boundary. The orientation of the tweezertrodes is more challenging due to the need to avoid significant current through the brainstem and thus fatality due to loss of vital function control. Therefore, we employed two methods (with equivalent success) that avoided any morbidity or lethality (Figure 2D, E). The first method involved contacting the lower third of the electrodes with the upper part of the hinge region of the animal’s head in presence of electrode gel (Figure 2D). Due to the natural curvature of the head, this drives current flow nearly tangential to the surface of the superior colliculus. The second method involved touching the lower third of the negative electrode to the back of the hinge of the neck and the positive electrode to the front of the nose, both with electrode gel (Figure 2E). Both methods led to similar results; specifically, a patch of electroporated cells roughly the same surface area as the superior colliculus (Figure 2F; 1.27 ± 0.11 mm^2^; *n* = 3). Again, similar numbers of cells were observed with both cytoplasmic EGFP and nuclear TagBFP reporter genes, and most cells labeled with both markers (Figure 2F, G). As with our results in the cortex, we never noted radial glial labeling at any time-point post-EP. Instead, cells appeared to migrate inward from 1 day post-EP (for example, see Figure 4A-A^2^ below) as compared with the more dispersed pattern observed 2 days following EP (Figure 2H).

**Figure 3:**
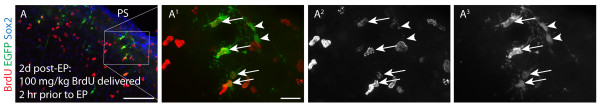
**Perinatal electroporation primarily targets proliferating cells.** (**A-A**^**3**^) After pre-pulsing animals with BrdU 2 h prior to EP, most EGFP^+^ cells are BrdU^+^ as shown by the arrows. Arrowheads denote two EGFP^+^ cells with lower but detectable amounts of BrdU. Note that labeled cells with similar amounts of BrdU seem to occur as doublets, suggesting that they are daughter cells derived from the same mitotic event. Scale bars measure 100 μm in (**A**) and 20 μm in (**A**^**1**^).

### Electroporated pial surface cells express neuronal and glial antigens

Using methodology similar to what we employed, EP has been found to selectively label proliferating CNS cells [17]. It is generally accepted that cells need to pass through M-phase within a window of about 8 h post-EP [17] (perhaps to allow for nuclear access of the plasmid DNA) in order to express the electroporated DNA. Furthermore, plasmid DNA was found to penetrate roughly 160 μm into CNS tissue [17]. Nonetheless, to empirically determine if this were the case, we labeled proliferating cells 2 h prior to EP as Stancik and colleagues had previously done in the developing cortex to ascertain whether we were targeting proliferating cells [17]. This allows for S-phase labeling of cells, some of which will be in M-phase at the time of EP. After 2 days, we found that 95 ± 1.1% of EGFP^+^ cells were BrdU^+^. In addition, cells with similar morphology and intensity of BrdU labeling were often found in doublets, suggesting that they might be clonally-related daughter cells (Figure 3A-A^3^).

**Figure 4:**
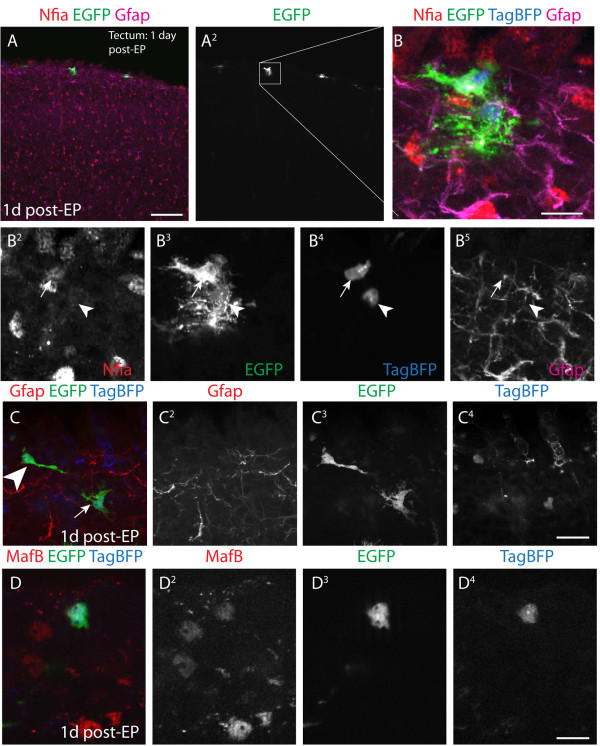
**Evaluation of cells 1 day following perinatal electroporation.** (**A-A**^**2**^) At 1 day post-EP, EGFP^+^ cells localize to the immediate pial surface. (**B-B**^**5**^) These cells often co-localize with the astroglial marker Nfia, weakly express Gfap, and display glial progenitor morphology. (**C-C**^**4**^) Morphology of EGFP^+^ cells is typically bipolar (arrowhead) or consists of a multipolar glial morphology (arrow). (**D-D**^**4**^) Bipolar or non-glial cells often express the interneuron marker MafB. Scale bars measure 100 μm in (**A**), 15 μm in (**B**)**,** 20 μm in (**C**^**4**^), and 10 μm in (**D**^**4**^).

To more precisely define the electroporated cell type, we examined cells at 1 day post-EP. As mentioned, compared with 2 days post-EP, these younger electroporated cells were located near to or even at the pial surface (Figure 4A-A^2^). Two morphologies consistent with neural progenitors were frequently noted in labeled cells. The first was consistent with a glial progenitor phenotype. These cells often labeled with the astroglial marker Nfia, and were weakly positive (and sometimes negative) for Gfap (Figure 4B-B^5^; Figure 4C, denoted by an arrow). The other electroporated cell type was bipolar or lacked processes (Figure 4C-C^4^, denoted by arrowhead) and expressed the *de facto* interneuron transcription factor, MafB (Figure 4D-D^4^). This antigen labels ventral forebrain-derived interneuron progenitors and interneurons throughout their life cycle, starting in the SVZ of the medial ganglionic eminence in the forebrain [18].

**Figure 5:**
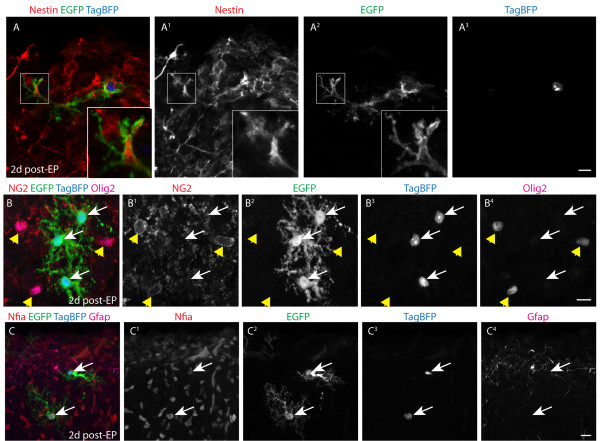
**Early expression of glial markers following perinatal electroporation.** (**A-A3**) Cells electroporated with membrane EGFP and TagBFP are Nestin^+^ after 2 days. Note the presence of Nestin within the membrane of the GFP^+^/TagBFP^+^ cell in the inset. (**B-B4**) EGFP^+^ (presumptive) glial cells did not stain for NG2. (**C-C4**) EGFP^+^ glial cells express Nfia and varying amounts of Gfap at 2 days post-EP. Scale bars measure 10 μm in (**A**^**3**^), (**B**^**4**^), and (**C**^**4**^).

To further characterize the populations of electroporated cells, we examined their expression of cell lineage markers at 2 days post-EP. First, we imaged these cells for Nestin, a marker of neural stem/progenitor cells. Because Nestin is a filamentous protein, a reporter plasmid that drives membrane EGFP expression was utilized to better identify co-localization. Numerous GFP^+^/TagBFP^+^ electroporated cells were identified that expressed filamentous Nestin protein (Figure 5A-A^3^). However, we did not detect NG2 expression by these cells (Figure 5B-B^4^). Although some of these newly electroporated cells expressed Olig2 (data not shown), astrocytic populations are actually known to express this antigen during development [19]. Similar to our findings at 1 day post-EP, 2-day cells had an immature astroglial morphology, frequently expressed Nfia, and had varying degrees of Gfap labeling, suggesting that they belonged to the astroglial lineage (Figure 5C-C^4^). Collectively, these data indicate that pial surface EP primarily targets neuronal and astroglial progenitors.

### Lineage assessment of pial surface electroporated cells

**Figure 6:**
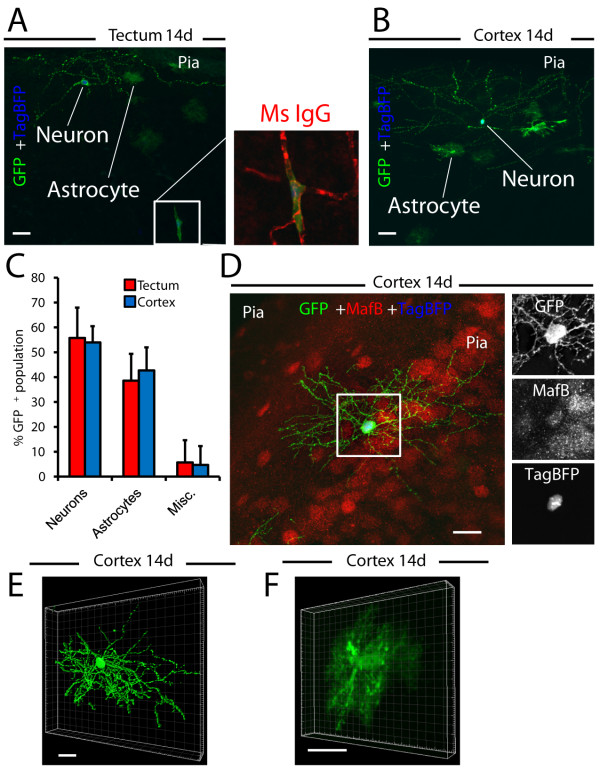
**Lineage assessment after perinatal electroporation.** (**A**) Cells electroporated with membrane EGFP and TagBFP are shown in the tectum, 14 days after electroporation. (**B**) Cells electroporated with membrane EGFP and TagBFP in the cerebral cortex are displayed, 14 days following electroporation. (**C**) Quantification of percentages of cells morphologically resembling neurons, astrocytes or miscellaneous populations is shown. (**D**) Electroporated cells with neuronal morphology express the interneuron-specific transcription factor, MafB. (**E**) Three-dimensional reconstruction and surface rendering of a GFP^+^ interneuron and (**F**) a GFP^+^ protoplasmic astrocyte using Imaris:Bitplane software. Scale bars measure 20 μm in A,B,D and 10 μm in E-F.

Following identification of electroporated neuronal and multipotent progenitors on the pial surface, we sought to determine their lineages. To achieve this, cells electroporated with membrane EGFP and nuclear TagBFP were morphologically assessed, 14 days post-EP. Interestingly, elaborate processes emanating from EGFP^+^ cells were noted that morphologically resembled immature interneurons on the surface of the tectum (Figure 6A) and cerebral cortex (Figure 6B). These cells were interspersed amongst EGFP^+^ cells morphologically resembling astrocytes, as well as a small fraction of miscellaneous cells that appeared to be pericytes or undefined glial cell types (Figure 6C). Morphological assessments were consistent with the notion that electroporated populations consisted primarily of cells from the neuronal (tectum, 55.74% ± 12.24%; cerebral cortex, 53.97% ± 6.53%) and astrocytic lineages (tectum, 38.58% ± 10.81%; cerebral cortex, 42.71% ± 9.27%). Cells of miscellaneous morphology (that is, pericytes, unclassified glia, or unidentifiable cells) comprised a small percentage of 14-day post-EP cells (tectum, 5.68% ± 8.94%; cerebral cortex, 4.71% ± 7.58%). To determine whether neuron-like 14-day post-EP cells were in fact interneurons, we immunostained for MafB. As demonstrated in Figure 6D, MafB expression co-localized with blue nuclear protein (TagBFP) within nuclei of EGFP^+^ neurons. It is important to note that neuronal and astroglial morphologies are quite different at this time point. Neurons have thin dendrites reaching hundreds of micrometers (Figure 6E), while astroglia form a tight cloud of fine processes with a diameter typically less than 50 μm (Figure 6F). Collectively, these data indicate that EP-targeted pial surface progenitors mature into cells phenotypically consistent with neurons and astrocytes. Moreover, some cells of neuronal morphology mature into MafB^+^ interneurons by 14 days post-EP.

### Differentiation of electroporated cells

**Figure 7:**
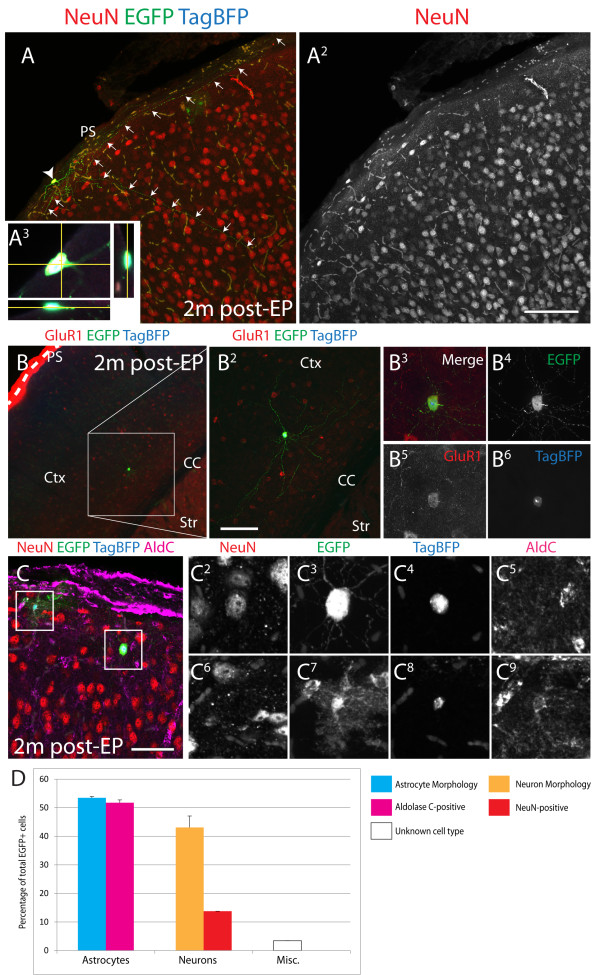
**Differentiation of perinatal electroporated cells in the cortex after 2 months.** (**A-A**^**2**^) An elongated cortical interneuron labeled with EGFP, NeuN, and TagBFP is shown at the pial surface, and an orthogonal view is displayed in (**A**^**3**^). (**B-B**^**6**^) A GluR1^+^ deep layer interneuron distal to the VZ and PS is shown. (**C-C**^**9**^) A NeuN^+^ neuron (**C**; **C**^**2**^**-C**^**5**^) and an Aldolase C^+^ astrocyte (**C**; **C**^**6**^**-C**^**9**^) are shown. (**D**) Quantification of EGFP^+^ cortical neurons and astrocytes is displayed at 2 months post-EP. Scale bars measure 100 μm in (**A**^**2**^) and (**B**^**2**^) and 50 μm in (**C**).

**Figure 8:**
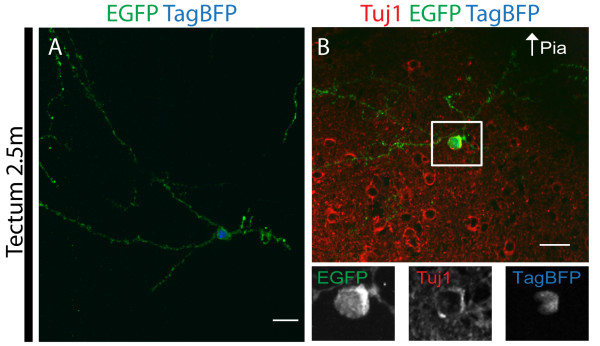
**Differentiation of pial surface cells in the superior colliculus 2.5 months after perinatal electroporation.** (**A**) A representative GFP^+^/TagBFP^+^ neuron with processes emanating from its soma is shown. Several processes measured hundreds of microns in length. (**B**) A representative GFP^+^/TagBFP^+^ cell that expresses the neuronal marker Tuj1 is shown. Scale bars measure 20 μm in (**A, B**).

Although the presence of cells morphologically representing neurons and astrocytes could be taken as evidence of maturation of electroporated pial surface progenitors, we sought to characterize the differentiation status of this population. To accomplish this, EP cells were analyzed 2 and 2.5 months post-EP, and compared to early (2 days post-EP) populations. Intricate neuronal processes were observed extending below the pial surface of the cerebral cortex 2 months post-EP in NeuN^+^ cells (Figure 7A-A^3^). In addition, many cells labeled for the interneuron marker GluR1 and could be found at sites distal from both the PS and VZ (that is, layers 5 to 6 of the cortex, Figure 7B-B^6^). We quantified numbers of cells with astroglial or neuronal morphology at 2 months, as well as the respective numbers of each population expressing Aldolase C or NeuN (as seen in Figure 7C-C^9^). Almost all presumptive astrocytes expressed the astrocyte marker Aldolase C (Figure 7D). However, only about one-third of cells with neuronal morphology expressed NeuN, a marker of differentiated neurons (Figure 7D). While the significance of this is unclear, lack of NeuN expression is not unique to this population, as this antigen does not label all neuronal subtypes [20]. For example, some neuronal populations in the piriform cortex also do not express NeuN [21,22]. In the tectum, 2.5-month post-EP GFP^+^/TagBFP^+^ cells appeared to have migrated inwards, dispersed, and differentiated in comparison to 2-day post-EP cells (Additional file 3: Figure 4). As demonstrated (in Figure 8A), some neuronal processes of GFP^+^/TagBFP^+^ cells measured several hundred microns in length at 2.5 months post-EP, demonstrating their maturation into stereotypical neurons. Finally, we immunostained 2.5-month post-EP GFP^+^/TagBFP^+^ populations with classical indicators of neuronal and astrocytic differentiation. Importantly, GFP^+^/TagBFP^+^ cells expressed the neuronal marker Tuj1 (Figure 8B), or alternately, the astrocytic marker Gfap (data not shown). These results demonstrate that cells targeted by EP are capable of differentiating into both neurons and astrocytes.

## Discussion

Over the past decade, EP has become a powerful tool for genetic manipulation of neural stem and progenitor cells during embryonic and postnatal development [12,23]. It has most often been utilized to target germinal zones surrounding the ventricles [13,15,16]. We have adapted and extended this technique to target the superficial layers of both the cortex and tectum at P2. In so doing, we provide a method for rapidly and specifically targeting these cell types. This technology will allow for elucidation of the genetic regulators of migration and differentiation of this population as well as their contribution to histogenesis during development and disease.

Our results demonstrate that postnatal EP can non-invasively target plasmids into the superficial progenitor domains rapidly and with a high degree of specificity. This methodology is given added importance, as we are not aware of any reporter mice or Cre recombinase lines capable of specifically targeting this unique pool of neural progenitors. Furthermore, while retroviruses have been used to specifically mark these cells [11], retroviral cloning and packaging is technically challenging and time-consuming. In addition, there are safety concerns associated with viral handling. EP simply requires purified plasmid DNA, which can be rapidly prepared. Furthermore, multiple genes or shRNAs can quickly be delivered, without the need for production of viral particles or complex mouse breeding strategies. However, it should be noted that the use of EP methodology and Cre-reporter or Cre-driver lines are not mutually exclusive.

We have provided a concise report of pial surface EP. The circumstantial evidence that we provide supports the progressive differentiation of immature neural progenitor populations into neurons and astroglia. Our results will need to be extended to rigorously determine the natural history of pial surface cell types and to exclude spurious or unexpected labeling mechanisms or transdifferentiation, which can be observed with such methods [12]. Thus, we envision that the electroporation of plasmids with cell type-specific promoter elements driving Cre, or conversely, the delivery of Cre-dependent reporter plasmids into cell-lineage dependent Cre lines, will be invaluable for the definitive determination of cell potential and lineage commitment at the pial surface.

EP is most easily performed during the perinatal period, due to visibility of brain structures and the injection site through the skull. Furthermore, this technique most efficiently targets proliferating cells that are actively undergoing breakdown of the nuclear envelope, ostensibly allowing for episomal expression of the plasmid [17]. Thus, during aging, EP efficiency would markedly decrease [14], as fewer and fewer cells are proliferating and targeting therefore becomes less accurate. Other potential caveats to EP include the episomal nature of most plasmids, whereby proliferation of cells may lead to plasmid dilution. Interestingly though, we did not observe much evidence of plasmid dilution within electroporated PS progenitors when compared to electroporated cells surrounding the lateral ventricles of the forebrain (data not shown). However, it did appear that astroglial EGFP expression declined between 14 days and 2 to 2.5 months post-EP, which is suggestive of further proliferation (data not shown). Nevertheless, these potential limitations could be overcome by using tools that allow for stable plasmid integration into the genome (for example, piggyBac as in [24], Tol2 [25], or Sleeping Beauty transposon systems).

Electroporation of the dorsal surface of the brain appears to target differentiating progenitor cells that initially do not express markers of mature neurons or astroglia. It is important to note that there are reports of *in vivo* postmitotic neuronal electroporation (reviewed in [23,26-28]). However, by and large, these electroporation approaches are of the more invasive microelectroporation variety, where electrodes are inserted into the tissue *vs.* the external application used in this report. Notably, our BrdU labeling experiments did not label 100% of electroporated cells, and only weakly labeled some cells. However, the cell cycle lengthens throughout the course of embryogenesis [29] and throughout postnatal life [30], making BrdU labeling less efficient. In addition, there are likely temporal and regional differences in cell cycle length between the postnatal pial surface and cortical VZ, where previous EP/BrdU double-labeling experiments were carried out [17,31]. Nevertheless, it is possible that we are targeting postmitotic populations, but these cells are likely to be immature, migrating, or otherwise in transition at the time of EP based on their progressive morphological differentiation. For example, EP of membrane-tagged EGFP demonstrated that the morphology of most cells was more undifferentiated, often in the form of multipolar glia or bipolar cells. At 14 days, cells displaying neuronal or astroglial morphologies did not strongly express the *de facto* markers of these respective cell types: NeuN (in both cortex and tectum) or Gfap (in the tectum only, as most non-activated cortical astrocytes do not normally express Gfap).

In our EP system, immunostaining and morphologic evidence of differentiated astroglia and neurons was present later on at 2 to 2.5 months. Even at this time, we did note that many cells with neuronal morphology did not label with NeuN. Notably, NeuN does not label all neuronal subtypes [20]. In particular, it has been shown that some neurons in the piriform cortex are negative for this antigen [22]. Based on available evidence then, we conclude that these cells represent a unique population of neurons that clearly display neuronal properties (thin dendrites stretching hundreds of μm, varicosities, and so on) and do not express non-neuronal lineage markers such as NG2, Olig2, or Aldolase C (data not shown). Interestingly, the longer time course of development of these unique pial progenitors seems to differ from typical VZ-derived neural cells. In summary, the perinatal EP technique detailed in this report should be amenable for rapid elucidation of the molecular mechanisms regulating this fascinating pial neural progenitor lineage.

## Conclusions

In this report, we present a perinatal EP method for rapid genetic labeling and genetic modification of marginal zone/layer I progenitors in dorsal regions of the brain. This population has only recently begun to be characterized, and the cell autonomous genetic regulation of these unique neural progenitors remains completely undetermined. EP will likely prove to be a valuable tool for further exploration of these important neurobiological questions.

## Methods

### Subcloning and plasmid preparation

Briefly, the Ufek (Ubiquitin C promoter driving farnesylated EGFP with Kozak site) plasmid was created by subcloning EGFP with a farnesylation domain from pEGFP-F (Clontech) into pUb-GFP (Plasmid 11155; Addgene) by In-fusion PCR subcloning. The pCagg-TagBFPnls construct was created by PCR subcloning the TagBFP coding sequence into pCAGEN with the inclusion of a V5 epitope tag and nuclear localization sequence in the carboxyl-terminal primer. Importantly, these plasmids lack nuclear import sequences associated with robust gene expression when transfected/electroporated into postmitotic cell types without transitioning through mitosis first [32]. Endotoxin-free plasmids were maxi-prepped using Nucleobond Xtra Maxi EF kits (Macherey-Nagel). Additional cloning details are available upon request.

### Postnatal electroporation

Postnatal day 2 mice were anesthetized by hypothermia. Injections were made free-hand using a pulled glass capillary pipette. During the injection, the capillary tube was used to penetrate the skin and skull. Using care, the tip was stopped from further penetration after first clearing the skull, which was indicated by the lack of further resistance felt just after piercing the skull. The left optic tectum was visually determined by locating lambda and then targeting the superior colliculus (just posterior and lateral to lambda) for injection. The skull was carefully pierced and 0.6 to 1.2 μL of plasmid solution containing fast green and 1.0 μg/μL of each plasmid was pressure-injected as in Figure 1A using a XenoWorks microinjection system. The cortex was similarly injected, but the injection was targeted to the center of the dorsal cortex as noted in Figure 2A. Care was taken to avoid the cerebral arteries, which might cause a local hematoma. Diagrams showing the injection strategy are shown in Additional file 1: Figure S1. Platinum tweezertrodes were then used to electroporate with three to five pulses of 135 V (50 ms; separated by 950 ms) generated using the ECM 830 BTX Electroporator (Harvard Apparatus). SignaGel was used to increase conductance. After EP, mice were placed on a heating pad prior to being returned to their cages. All animal experiments were performed in accordance with NIH guidelines, and were approved by the Cedars-Sinai Medical Center Institutional Animal Care and Use Committee under protocol IACUC003507.

### BrdU labeling

In order to label dividing neural cells, pups were i.p. injected with 100 mg/kg of BrdU dissolved in sterile saline (10 mg/mL). This procedure was carried out 2 h prior to EP.

### Tissue processing

Electroporated pups were collected at defined postnatal ages, and their brains were immediately dissected in PBS and fixed in 4% PFA 6 h to overnight at 4 °C. On the following day, brains were embedded in 4% LMP agarose, sectioned at 70 or 250 μm thickness on a Leica VT1000S vibratome and then processed for immunohistochemistry.

### Immunohistochemistry

Sections were reacted with various primary antibodies (diluted in PBS containing 10% normal donkey serum and 0.03% Triton-X) overnight at 4 °C. (For BrdU immunohistochemistry, tissue was treated with 2 N HCL for 15 min at 37 °C prior to immunostaining). Primary antibodies were detected using fluorescent-conjugated secondary antibodies (Jackson Immunobiology) diluted at 1:1,000 in PBS containing 0.03% Triton-X. The following primary antibodies were used: goat anti-Aldolase C (1:300; Santa Cruz), rat anti-BrdU (1:250; Accurate), goat anti-Olig2 (1:500; R&D Systems), goat anti-Dcx (1:500; Santa Cruz Biotechnology), mouse anti-Nestin (1:2,000; Abcam), chicken anti-EGFP (1:5,000; Abcam), guinea pig anti-Gfap (1:1,000; Synaptic Systems), rabbit anti-GluR1 (1:500; Millipore), rabbit anti-MafB (1:250-1,000; Bethyl Laboartories), mouse anti-NeuN (1:200; Millipore), rabbit anti-Nfia (1:1,000; active motif), rabbit anti-NG2 (1:250; Millipore), goat anti-Sox2 (1:500; Santa Cruz), mouse anti-Tuj1 (1:1,000; Sigma), rabbit anti-GFAP (1:2,000; Dako), and mouse anti-V5 (1:2,000; Life Technologies). All images were acquired on either a Zeiss Apotome, a Nikon A1R confocal, or a Nikon AZ-C1 macroconfocal microscope in independent fluorescence channels.

### Image analysis

Electroporated cells were counted using ImageJ, release 1.45. The Nikon AZ-C1 macroconfocal was employed for quantification in Figures 1 and 2. All other quantification of electroporated populations was performed on images acquired with a Nikon A1R confocal microscope. Three images per region were acquired using a 20X objective and quantified using Nikon Elements, release 3.2. For 1 and 2 day quantifications, cells displaying apoptotic characteristics (pyknotic nuclei, membrane blebbing, detached processes) were disregarded. For lineage assessments, a total of 50 to 100 cells per region were quantified from 2-3 animals. Data represent averages of three sections from two to three animals.

## Abbreviations

Cb, Cerebellum; Dcx, Doublecortin; EGFP, Enhanced green fluorescent protein; EGL, External granule layer; EP, Electroporation; GFP, Green fluorescent protein; L-Ctx, Left cortex; IUE, In utero electroporation; IZ, Intermediate zone; LH, Left hemisphere; MZ, Marginal zone; P, Postnatal; R-Ctx, Right cortex; RH, Right hemisphere; SC, Superior colliculus; SVZ, Subventricular zone; TagBFP, Blue nuclear protein.

## Competing interests

The authors declare that they have no competing interests.

## Authors’ contributions

JJB, DG, and TT designed the experiments. JJB, DG, RL, GK, and JR performed all experiments. JJB, DG, and RL contributed unique tools/analytical reagents. JJB, DG, and TT wrote and JJB, DG, CNS, and TT edited the manuscript. All authors read and approved the final manuscript.

## Supplementary Material

Additional file 1 **Figure S1.** Diagrams are shown depicting the injection procedure for (**A**) cortical and (**B**) tectal delivery of plasmid DNA solution. The solution was delivered at the level of the pia mater, roughly at the surface of the parenchyma. Click here for file

Additional file 2 **Figure S2.** Injection of plasmid solution into the ventricles leads to EGFP^+^ labeling of radial glia processes when the electrode orientation used for pial surface perinatal electroporation is employed. (**A-A**^**4**^) Radial glia oriented into the hippocampus and toward the midline are the predominant populations labeled by a current directed across the pial surface (the orientation typically used for pial surface EP) when plasmid DNA is delivered into the ventricles (approximate location denoted by an asterisk), rather than at the pial surface. Note the prominent radial glial end-feet at the medial cortex and lack of cell bodies observed in (**A**^**4**^). Scale bar measures 200 μm in (**A**^**3**^). Click here for file

Additional file 3 **Figure S3.** Electroporated populations of tectum cells are shown at 2 days and at 2.5 months following perinatal electroporation. Note the presence of neuronal processes running below the pial surface and the dispersed TagBFP^+^ nuclei in electroporated cells. Scale bars measure 100 μm. Click here for file
