# Supplementary figures and images for: Rapid genetic targeting of pial surface neural progenitors and immature neurons by neonatal electroporation
Source: Neural Dev. 2012 Jul 10;7:26. doi: 10.1186/1749-8104-7-26 (PMC3479020; doi:10.1186/1749-8104-7-26)

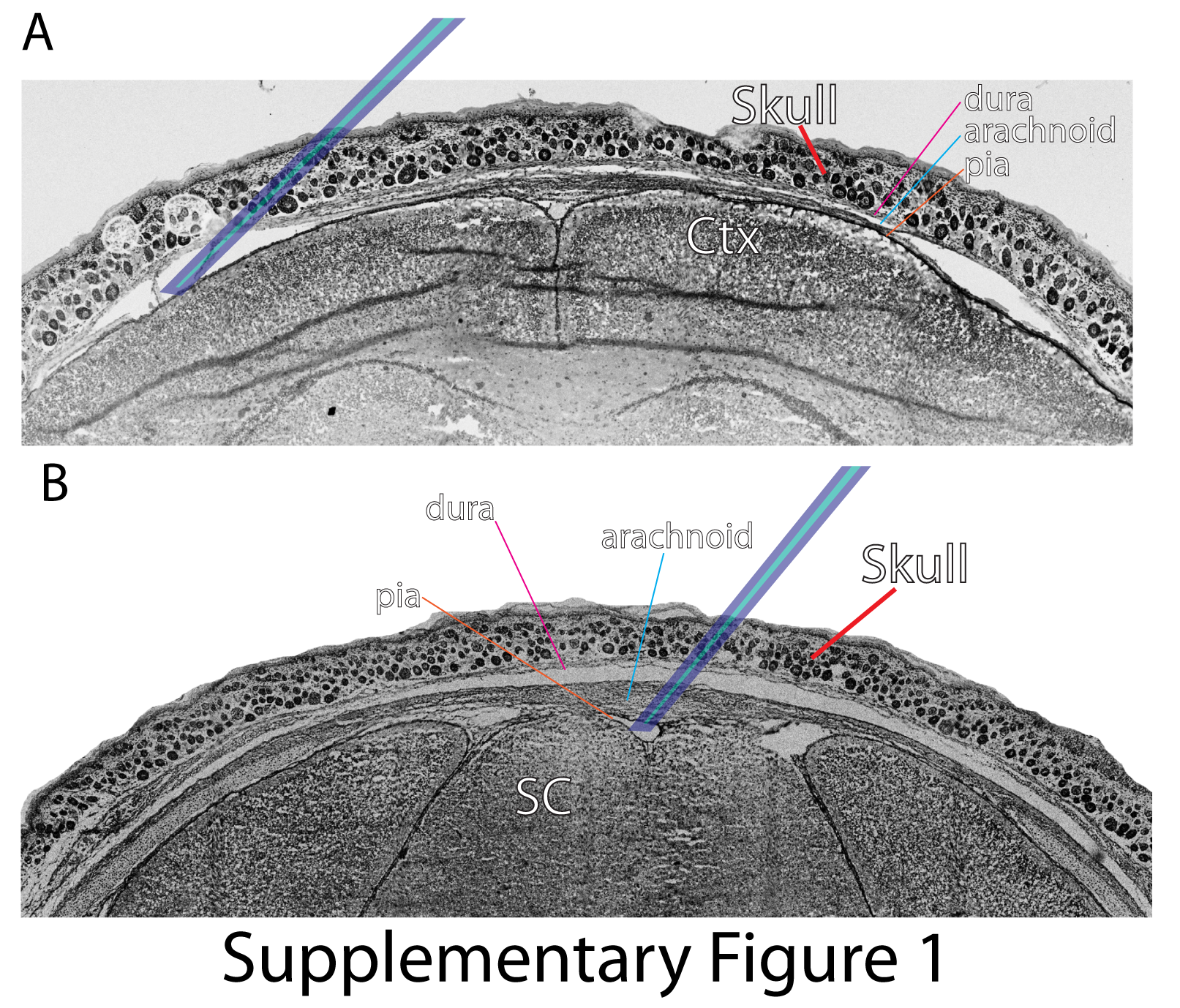

Supplement: Additional file 1 — Figure S1. Diagrams are shown depicting the injection procedure for (A) cortical and (B) tectal delivery of plasmid DNA solution. The solution was delivered at the level of the pia mater, roughly at the surface of the parenchyma. [file 1749-8104-7-26-S1.tiff]

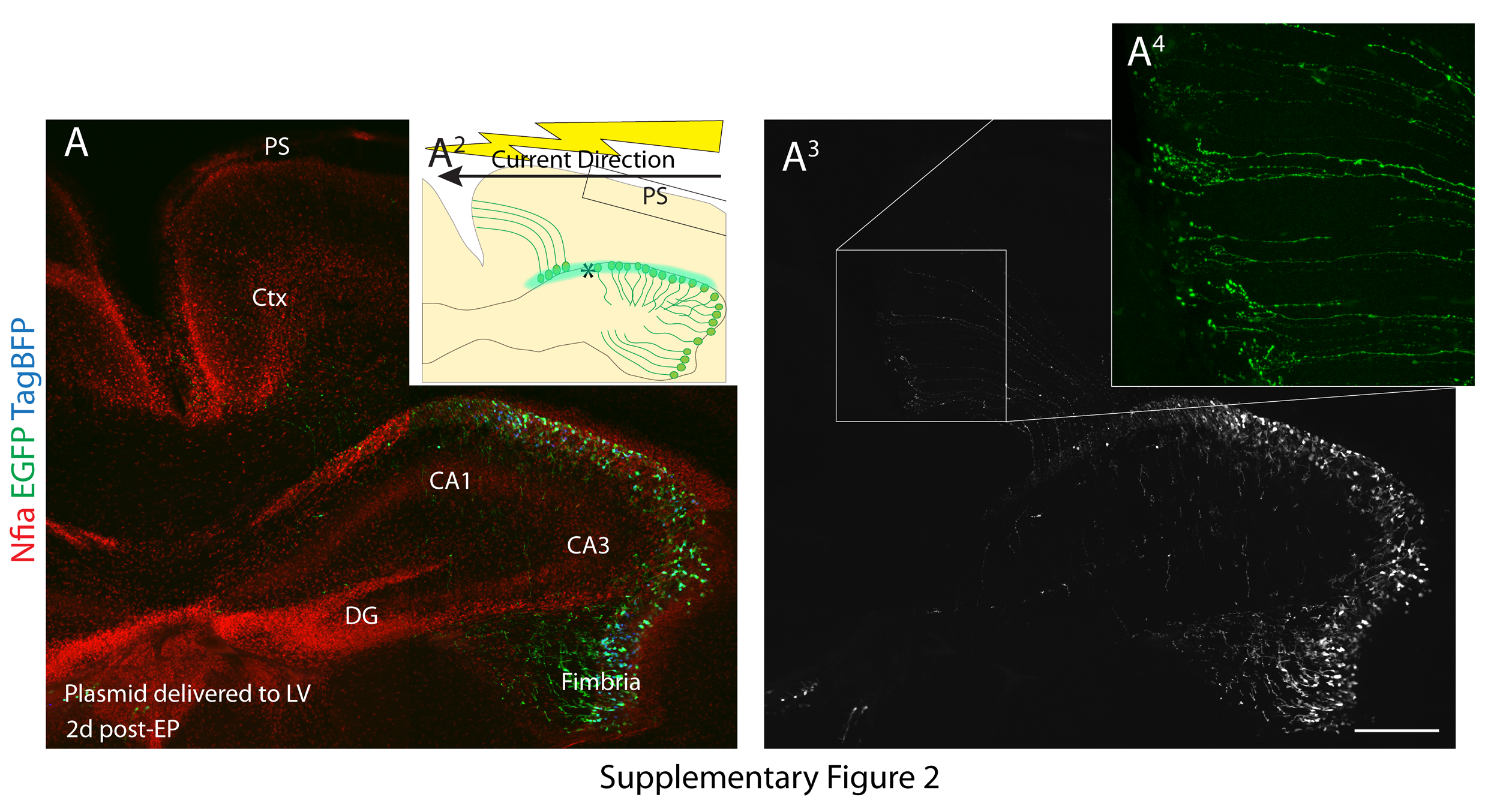

Supplement: Additional file 2 — Figure S2. Injection of plasmid solution into the ventricles leads to EGFP+ labeling of radial glia processes when the electrode orientation used for pial surface perinatal electroporation is employed. (A-A4) Radial glia oriented into the hippocampus and toward the midline are the predominant populations labeled by a current directed across the pial surface (the orientation typically used for pial surface EP) when plasmid DNA is delivered into the ventricles (approximate location denoted by an asterisk), rather than at the pial surface. Note the prominent radial glial end-feet at the medial cortex and lack of cell bodies observed in (A4). Scale bar measures 200 μm in (A3). [file 1749-8104-7-26-S2.tiff]

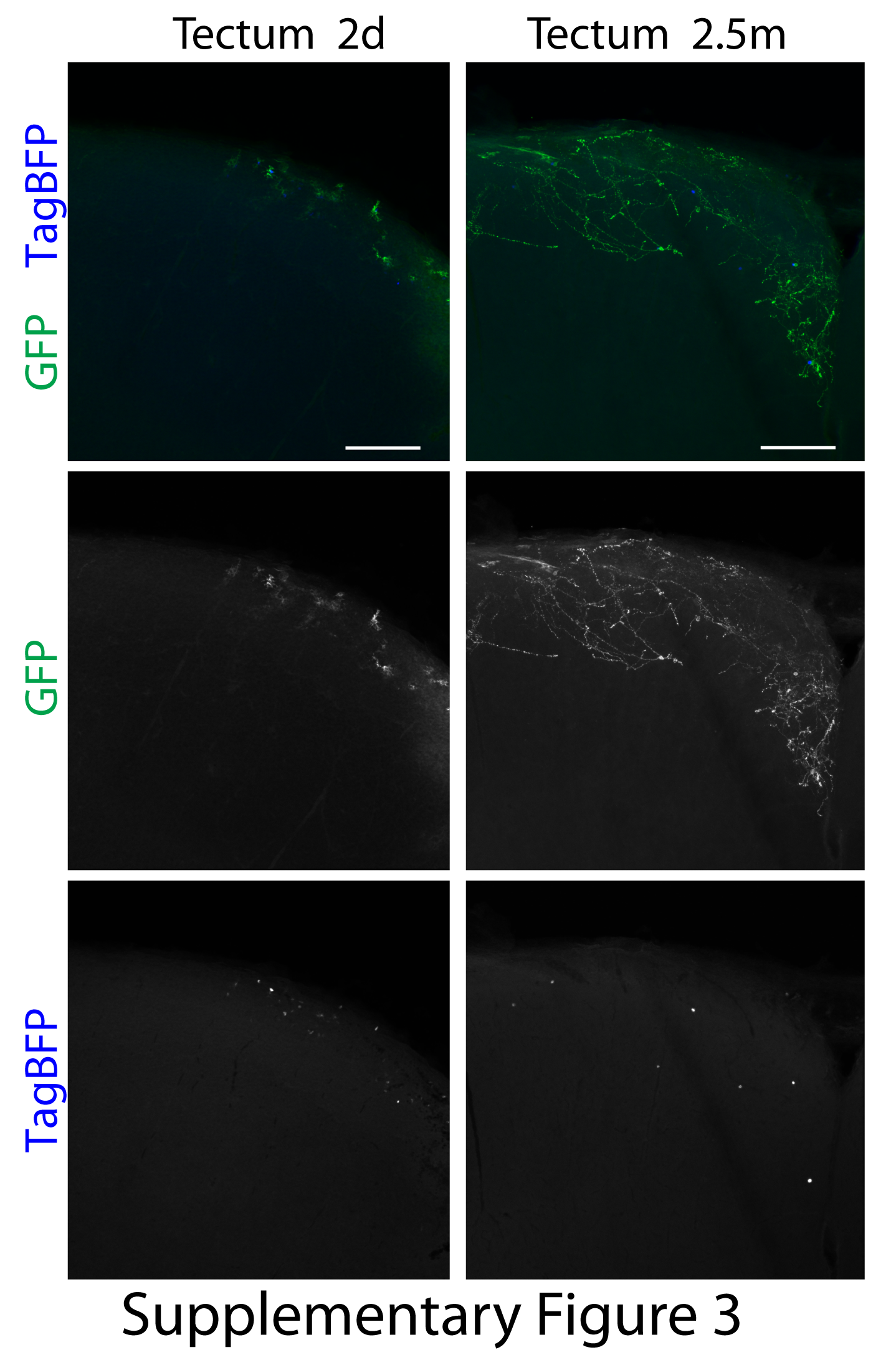

Supplement: Additional file 3 — Figure S3. Electroporated populations of tectum cells are shown at 2 days and at 2.5 months following perinatal electroporation. Note the presence of neuronal processes running below the pial surface and the dispersed TagBFP+ nuclei in electroporated cells. Scale bars measure 100 μm. [file 1749-8104-7-26-S3.tiff]
